# Recreational use of nitrous oxide causes seizure, pneumothorax, pneumomediastinum, and pneumopericardium: nitrous oxide and its harm, a case report

**DOI:** 10.48101/ujms.v128.10281

**Published:** 2023-12-08

**Authors:** Lee Ti Davidson

**Affiliations:** a Department of Emergency Medicine, Linköping University, Linköping, Sweden; b Department of Biomedical and Clinical Sciences, Linköping University, Linköping, Sweden

**Keywords:** Pneumothorax, pneumopericardium, recreational drug, nitrous oxide

## Abstract

Nitrous oxide, commonly known as ‘laughing gas’, has become a popular recreational drug. Whippets, small canisters containing gas in pressurized form, can be easily obtained from a food store. However, inhaling nitrous oxide from these canisters, which contain a 100% concentration, can lead to hypoxia, resulting in seizures or even death. Inhalation of nitrous oxide rarely causes pneumothorax, pneumomediastinum, and pneumopericardium.

This case study highlights the potential dangers of recreational abuse of nitrous oxide.

## Introduction

The use of nitrous oxide as a recreational drug has significantly increased worldwide over the past decade (1), and it is now a common occurrence in Sweden. Nitrous oxide is easily accessible and is primarily used as a propellant in cream dispensers. Nitrous oxide cartridges can be legally purchased from party stores and online. A common misconception is that nitrous oxide is harmless because it is used in health care and is legal in Sweden. The Swedish Poison Information Center states that the most common method of consuming nitrous oxide for recreational purposes is inhaling the gas from balloons, less commonly directly into the mouth.

This case study highlights the potential for harm posed by recreational abuse of this substance.

We obtained written informed consent from the patient; single case reports are exempt from institutional review board evaluation.

## Case presentation

This is the case of a 25-year-old man, a non-smoker with no known medical conditions or drug abuse, who presented to the emergency department (ED) complaining of chest pain. He reported consuming 20 centiliters of whisky and inhaling nitrous oxide directly from its canisters, using a device designed for dispensing whipped cream. After inhaling for the fourth time, he collapsed and experienced a brief generalized seizure. An ambulance was called, and he was immediately transported to the ED. His seizures ceased spontaneously within 5 min, and he regained consciousness but complained of severe chest pain, especially while breathing. He did not report vomiting or any other form of trauma. During ambulance transport, he was given a 4 mg dose of morphine intravenously (IV) and 15 liters of oxygen. He was also receiving a continuous infusion of Ringer acetate. Upon arrival at the ED, his chest pain had improved, and his vital signs were normal.

Upon examination, there was no airway compromise. Still, the patient exhibited discreet subcutaneous crepitation at the suprasternal notch and on the medial right clavicle area, indicative of subcutaneous emphysema. Additionally, the patient showed reduced lung percussion on the right side and a bubbling sound with auscultation on the anterior chest over the precordial area. His neurological status was found to be normal.

**Figure 1:**
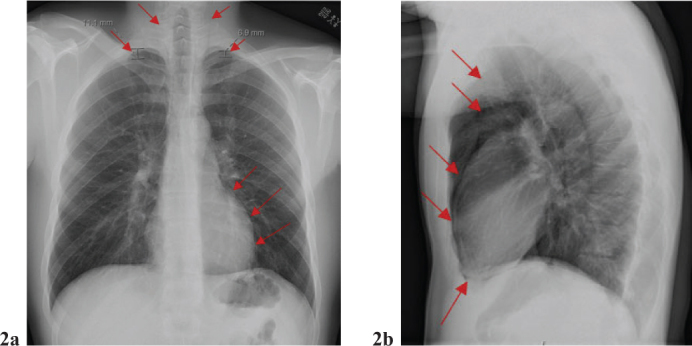
X-ray of the lungs: There was a suspicion of bilateral pneumothorax, with a gap of about 1 cm on the right side and 7 mm on the left side at the apex. Additionally, air was seen around the heart, in the mediastinum, and up the neck. This is not a typical picture, but there may be pneumopericardium and pneumomediastinum. The radiologist suggested further tests would be needed to confirm the findings.

The electrocardiograph (ECG) showed normal readings, except for a sinus tachycardia of around 130 beats per minute. A bedside ultrasound on the right anterior chest revealed an absence of normal lung sliding and the presence of the Barcode sign. A chest X-ray indicated the presence of bilateral pneumothorax and pneumomediastinum (Figure 1a–b).

**Figure 2:**
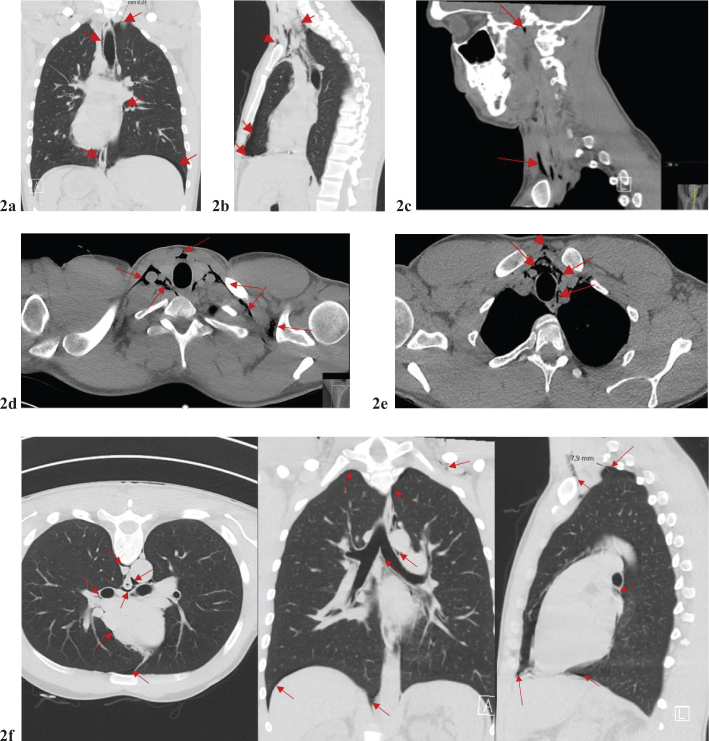
(a–f) Chest CT: As with the previous chest X-ray, a bilateral pneumothorax was confirmed. *Pronounced pneumomediastinum extending up the* neck bilaterally. Air (red arrow) has also been dissected along large bronchi and to the pericardium.

Upon reevaluation of the physical examination, it was found that the subcutaneous emphysema had progressed to the supraclavicular region, and the patient complained of pain in the left shoulder. A subsequent computed tomography of the chest confirmed bilateral pneumothorax, pneumomediastinum with air extending up to the neck, pneumopericardium, and pneumoperitoneum (Figure 2a–f). Additionally, the air was observed dissecting along the major bronchus toward the pericardium (Figure 2f). A small amount of air was noted to have dissected its way up to the neck, right up to the base of the skull (Figure 2a–e). Most air is in the lower throat. After consulting a surgeon and conducting further investigations, it was determined that esophageal perforation was not the cause of the issue.

**Figure 3:**
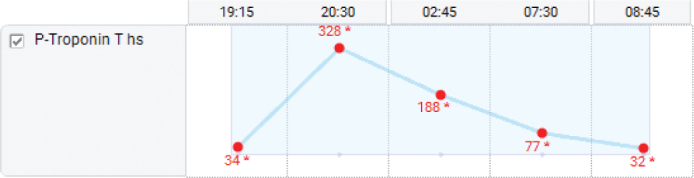
Level of plasma highly sensitive troponin on arrival at the emergency department to the next day.

The urine dipstick test revealed a trace of cocaine. Upon further questioning, the patient disclosed the recent use of a smaller amount of the drug. The blood test indicated increased high-sensitive troponin levels, ranging 34 to 328 (Figure 3), while the repeated ECG was normal.

While at the ED, he was given an additional dose of morphine and paracetamol through an IV. He reported no subsequent pain and was prescribed oral paracetamol every 6 h. The amount of administered oxygen decreased to 4 liters, and his pulse oximetry reading indicated a peripheral oxygen concentration of 100%. He was initially admitted to the intensive care unit for observation and transferred to the cardiothoracic department the day after. The cardiac ultrasound was normal, as was the rest of the medical observation. After a thorough review of his case, a team of doctors, including a surgeon, cardiologist, and thoracic surgeon, have concluded that his condition was caused by barotrauma resulting from inhaling nitrous oxide from a cream dispenser. They opted for a conservative management approach, and he was discharged following a period of clinical stability with an uneventful recovery.

## Discussion

Recreational use of nitrous oxide has become widespread since 2000 (1). According to the Central Association for Alcohol and Drug Information’s (CAN) 2022 annual national school survey in Sweden, 6% of grade nine students reported trying nitrous oxide, while the corresponding figure for year 2 high-school students was 17%. The survey also revealed that nitrous oxide use is more prevalent in urban areas, equally common among boys and girls, and strongly associated with alcohol, drugs, and tobacco (2).

Nitrous oxide is commonly used in healthcare to provide short-term sedation during painful, uncomfortable, or anxiety-inducing procedures. Its anesthetic effect is achieved by inhibiting N-methyl-D-aspartate (NMDA)-type glutamate receptors in the central nervous system. Nitrous oxide releases endogenous opioids, like morphine, which provides pain relief. Additionally, it activates γ-aminobutyric acid (GABA) type A receptors, leading to anxiolysis (3, 4).

In health care, it is common practice to administer 100% oxygen to patients for a minimum of 5 min before and after the administration of nitrous oxide. This is done to prevent hypoxia. Administering pure oxygen results in high oxygen concentrations, which increases the pulmonary oxygen reservoir and prevents the development of hypoxic gas mixtures within the breathing system, improving patients’ safety (5). Nitrous oxide is delivered with oxygen through a continuous flow mixer that uses at least 30% oxygen to avoid hypoxemia. The concentration of nitrous oxide can be adjusted between 30 and 70% (6). A pre-mixed combination of 50% nitrous oxide and 50% oxygen, known as Entonox^®^, can be used for analgesia on a patient-controlled analgesia or demand-triggered system. Administration of up to 70% concentrations of nitrous oxide, combined with oxygen, is safe (7). The nitrous oxide delivery system collects the patient’s exhaled breath to ensure the safety of doctors and clinic staff. Procedure rooms are equipped with ventilation systems that maintain a steady flow of fresh air, thus preventing long-term nitrous oxide exposure for medical staff.

Although nitrous oxide is often perceived as harmless (8), incorrect utilization poses serious short- and long-term health threats, especially in the young population. Nitrous oxide, which young people often abuse, is generally more concentrated than its clinical counterpart. This higher concentration of nitrous oxide can produce a short-lived yet intense state of intoxication that typically lasts for approximately 1–2 min. This state is characterized by heightened euphoria, relaxation, and, occasionally, hallucinations. Furthermore, users experience a degree of pain relief upon inhalation. However, this concentration of nitrous oxide depresses the sensation of dyspnea, and users may succumb to hypoxia without realizing the immediate danger (9).

Nitrous oxide has high solubility, which can cause the expansion of closed gas-filled cavities in the body. When nitrous oxide from the blood replaces small volumes of nitrogen in the cavity, it enlarges the cavity. This can impair cerebral blood flow-activity coupling (10) and worsen air space conditions such as pneumothorax, air embolism, and bowel distension (11, 12). High doses of nitrous oxide can cause hypoxia, arrhythmias, myocardial infarction (13), malignant hyperthermia, seizures, pneumothorax, pneumomediastinum, and subcutaneous emphysema (14). An experiment study found that 25% nitrous oxide with inhaled air depressed dyspnea sensation. At 50% concentrations, test persons did not experience dyspnea despite rapid arterial oxygen desaturation (9). Unfortunately, fatalities due to sudden cardiac arrhythmias or asphyxiation following nitrous oxide use are rarely reported (15, 16).

In its concentrated form, nitrous oxide can be very cold and cause frostbite injury. The injuries can occur by direct contact with the compressed canister or inhalation (17–19). Acute nitrous oxide inhalation can cause frostbite to the oral cavity and upper aerodigestive tract, respiratory irritation, and airway compromise due to edema. Edema and necrosis of the airway may require intubation or tracheotomy of the patient and can lead to long-term complications (20).

Repeated or prolonged use of nitrous oxide can lead to irreversible neurological damage by inhibiting the activity of the vitamin B12-dependent enzyme methionine synthase (11, 21). This can result in symptoms such as loss of feeling; numbness; reduced strength in fingers, hands, arms, and legs; and difficulty urinating, standing, and walking. Additionally, individuals may experience anxiety, psychosis, and cognitive impairment like dementia (22, 23). This condition may also occur in individuals with undiagnosed cobalamin deficiency, such as vegans, pernicious anemia, and hereditary disorders of cobalamin and folate metabolism. In rare cases, repeated exposure to nitrous oxide can lead to subacute combined degeneration of the spinal cord (SACD) and even death, as seen in a congenital 5,10-methylenetetrahydrofolate reductase (MTHFR) deficiency case (21).

Some less-known side effects of nitrous oxide abuse are various thromboembolic complications inclusive of major adverse cardiac and vascular events, especially in young patients without typical cardiovascular risk factors (24). The underlying pathophysiological mechanism is not yet fully understood but may be related to the interaction of the abuse of nitrous oxide with vitamin B12, a cofactor of the methionine synthase complex, leading to an increase in homocysteine levels. High levels of homocysteine are associated with dysfunction of the endothelium, oxidative stress, increased platelet activation, higher production of thrombin, enhanced activity of factor V, impaired fibrinolysis, and inflammation of the blood vessels leading to early development of atherosclerosis, arteriosclerosis, and thromboembolism (25). Elevated homocysteine levels are showed to be associated with arterial thrombus formation, peripheral arterial disease, ischemic heart disease and myocardial infarction, stroke, pulmonary embolism, and thrombus formation in the abdominal veins (13, 24–29).

This patient inhaled a high concentration of nitrous oxide directly from a canister. This caused cerebral hypoxia and a sudden change in intra-alveolar pressure due to forced lung expansion. As a result, the patient experienced seizures, and there was a rapid expansion of free air in the mediastinum, which permeated to all surrounding structures, causing pneumopericardium, pneumothorax, pneumomediastinum, and subcutaneous emphysema. The highly sensitive troponin was significantly elevated, an indication of myocardial injury, but the ECG and cardiac ultrasound did not indicate signs of infarction. This was likely due to hypoxia-induced myocardial injury and increased metabolic demand by the generalized seizure, although transient hyperhomocysteinemia related to nitrous oxide abuse could not be excluded. Unfortunately, there was no record of the patient’s homocysteine levels. The possibility that cocaine contributed to the elevation of troponin could not be excluded despite the patient denying its consumption on the actual occasion.

## References

[1] van Amsterdam JG, Nabben T, van den Brink W. Increasing recreational nitrous oxide use: should we worry? A narrative review. J Psychopharmacol. 2022;36(8):943–50. doi: 10.1177/02698811221082442 35678512

[2] Guttormsson Ulf GI. Lustgas bland unga 2022. Stockholm: Centralförbundet för alkohol- och narkotikaupplysning; 2022.

[3] Emmanouil DE, Quock RM. Advances in understanding the actions of nitrous oxide. Anesth Prog. 2007;54(1):9–18. doi: 10.2344/0003-3006(2007)54[9:Aiutao]2.0.Co;2 17352529 PMC1821130

[4] Sanders RD, Weimann J, Maze M. Biologic effects of nitrous oxide: a mechanistic and toxicologic review. Anesthesiology. 2008;109(4):707–22. doi: 10.1097/ALN.0b013e3181870a17 18813051

[5] Baum JA. The carrier gas in anaesthesia: nitrous oxide/oxygen, medical air/oxygen and pure oxygen. Curr Opin Anaesthesiol. 2004;17(6):513–16. doi: 10.1097/00001503-200412000-00012 17031087

[6] Huang C, Johnson N. Nitrous oxide, from the operating room to the emergency department. Curr Emerg Hosp Med Rep. 2016;4:11–18. doi: 10.1007/s40138-016-0092-3 27073749 PMC4819505

[7] Babl FE, Oakley E, Seaman C, Barnett P, Sharwood LN. High-concentration nitrous oxide for procedural sedation in children: adverse events and depth of sedation. Pediatrics. 2008;121(3):e528–32. doi: 10.1542/peds.2007-1044 18310173

[8] Kaar SJ, Ferris J, Waldron J, Devaney M, Ramsey J, Winstock AR. Up: the rise of nitrous oxide abuse. An international survey of contemporary nitrous oxide use. J Psychopharmacol. 2016;30(4):395–401. doi: 10.1177/0269881116632375 26912510

[9] Bäckström B, Johansson B, Eriksson A. Death from nitrous oxide. J Forensic Sci. 2015;60(6):1662–5. doi: 10.1111/1556-4029.12879 26258592

[10] Kaisti KK, Långsjö JW, Aalto S, Oikonen V, Sipilä H, Teräs M, et al. Effects of sevoflurane, propofol, and adjunct nitrous oxide on regional cerebral blood flow, oxygen consumption, and blood volume in humans. Anesthesiology. 2003;99(3):603–13. doi: 10.1097/00000542-200309000-00015 12960544

[11] Myles PS, Leslie K, Silbert B, Paech MJ, Peyton P. A review of the risks and benefits of nitrous oxide in current anaesthetic practice. Anaesth Intens Care. 2004;32(2):165–72. doi: 10.1177/0310057x0403200202 15957712

[12] Akca O, Lenhardt R, Fleischmann E, Treschan T, Greif R, Fleischhackl R, et al. Nitrous oxide increases the incidence of bowel distension in patients undergoing elective colon resection. Acta Anaesthesiol Scand. 2004;48(7):894–8. doi: 10.1111/j.0001-5172.2004.00427.x 15242436 PMC1351324

[13] Oomens T, Riezebos RK, Amoroso G, Kuipers RS. Case report of an acute myocardial infarction after high-dose recreational nitrous oxide use: a consequence of hyperhomocysteinaemia? Eur Heart J Case Rep. 2021;5(2):ytaa557. doi: 10.1093/ehjcr/ytaa557 33598625 PMC7873791

[14] McDermott R, Tsang K, Hamilton N, Belton M. Recreational nitrous oxide inhalation as a rare cause of spontaneous pneumomediastinum. BMJ Case Rep. 2015;2015:bcr2015209750. doi: 10.1136/bcr-2015-209750 PMC4577686 26392441

[15] Wagner SA, Clark MA, Wesche DL, Doedens DJ, Lloyd AW. Asphyxial deaths from the recreational use of nitrous oxide. J Forensic Sci. 1992;37(4):1008–15. doi: 10.1520/JFS13286J 1506823

[16] Drug-related deaths involving nitrous oxide in England and Wales, 1993 to 2017. London: Office for National Statistic (ONS); 2018.

[17] Quax MLJ, Van Der Steenhoven TJ, Antonius Bronkhorst MWG, Emmink BL. Frostbite injury: an unknown risk when using nitrous oxide as a party drug. Acta Chir Belg. 2022;122(2):140–3. doi: 10.1080/00015458.2020.1782160 32543291

[18] Chen JHC, Eriksson S, Mohamed H, Bhatti S, Frew Q, Barnes D. Experiences of frostbite injury from recreational use of nitrous oxide canisters in a UK burns center: not a laughing matter. J Plast Reconstr Aesthet Surg. 2023;83:282–8. doi: 10.1016/j.bjps.2023.05.012 37290369

[19] Rowson AC, Yii MX, Tan HB, Prasad J. Recreational nitrous oxide-induced injury to the soft palate. Clin Case Rep. 2023;11(9):e7858. doi: 10.1002/ccr3.7858 37649901 PMC10462773

[20] Chan SA, Alfonso KP, Comer BT. Upper aerodigestive tract frostbite from inhalation of automotive nitrous oxide. Ear Nose Throat J. 2018;97(9):E13–14. doi: 10.1177/014556131809700903 30273435

[21] Buhre W, Disma N, Hendrickx J, DeHert S, Hollmann MW, Huhn R, et al. European Society of Anaesthesiology Task Force on Nitrous Oxide: a narrative review of its role in clinical practice. Br J Anaesth. 2019;122(5):587–604. doi: 10.1016/j.bja.2019.01.023 30916011

[22] Xiang Y, Li L, Ma X, Li S, Xue Y, Yan P, et al. Recreational nitrous oxide abuse: prevalence, neurotoxicity, and treatment. Neurotox Res. 2021;39(3):975–85. doi: 10.1007/s12640-021-00352-y 33770366

[23] Redmond J, Cruse B, Kiers L. Nitrous oxide-induced neurological disorders: an increasing public health concern. Intern Med J. 2022;52(5):740–4. doi: 10.1111/imj.15544 34569693

[24] Oulkadi S, Peters B, Vliegen AS. Thromboembolic complications of recreational nitrous oxide (ab)use: a systematic review. J Thromb Thrombolysis. 2022;54(4):686–95. doi: 10.1007/s11239-022-02673-x 35759070

[25] Pushpakumar S, Kundu S, Sen U. Endothelial dysfunction: the link between homocysteine and hydrogen sulfide. Curr Med Chem. 2014;21(32):3662–72. doi: 10.2174/0929867321666140706142335 25005183 PMC5539954

[26] Fu Y, Wang X, Kong W. Hyperhomocysteinaemia and vascular injury: advances in mechanisms and drug targets. Br J Pharmacol. 2018;175(8):1173–89. doi: 10.1111/bph.13988 28836260 PMC5867019

[27] Liu F, Du J, Nie M, Fu J, Sun J. 5,10-methylenetetrahydrofolate reductase C677T gene polymorphism and peripheral arterial disease: a meta-analysis. Vascular. 2021;29(6):913–19. doi: 10.1177/1708538120982698 33357155

[28] Homocysteine and risk of ischemic heart disease and stroke: a meta-analysis. JAMA. 2002;288(16):2015–22. doi: 10.1001/jama.288.16.2015 12387654

[29] Vollenbrock SE, Fokkema TM, Leijdekkers VJ, Vahl AC, Konings R, van Nieuwenhuizen RC. Nitrous oxide abuse associated with severe thromboembolic complications. Eur J Vasc Endovasc Surg. 2021;62(4):656–7. doi: 10.1016/j.ejvs.2021.05.041 34275729

